# Integrative quantitative-phase and airy light-sheet imaging

**DOI:** 10.1038/s41598-020-76730-x

**Published:** 2020-11-19

**Authors:** N. R. Subedi, P. S. Jung, E. L. Bredeweg, S. Nemati, S. E. Baker, D. N. Christodoulides, A. E. Vasdekis

**Affiliations:** 1 Department of Physics, University of Idaho, Moscow, ID 83844 USA; 2 CREOL-The College of Optics and Photonics, University of Central Florida, Orlando, FL 32816-2700 USA; 3 Faculty of Physics, Warsaw University of Technology, Warsaw, Poland; 4 Environmental Molecular Sciences Laboratory, Pacific Northwest National Laboratory, Richland, WA 99354 USA

**Keywords:** Optical imaging, Interference microscopy, Light-sheet microscopy, Cellular noise

## Abstract

Light-sheet microscopy enables considerable speed and phototoxicity gains, while quantitative-phase imaging confers label-free recognition of cells and organelles, and quantifies their number-density that, thermodynamically, is more representative of metabolism than size. Here, we report the fusion of these two imaging modalities onto a standard inverted microscope that retains compatibility with microfluidics and open-source software for image acquisition and processing. An accelerating Airy-beam light-sheet critically enabled imaging areas that were greater by more than one order of magnitude than a Gaussian beam illumination and matched exactly those of quantitative-phase imaging. Using this integrative imaging system, we performed a demonstrative multivariate investigation of live-cells in microfluidics that unmasked that cellular noise can affect the compartmental localization of metabolic reactions. We detail the design, assembly, and performance of the integrative imaging system, and discuss potential applications in biotechnology and evolutionary biology.

## Introduction

Selective plane illumination microscopy (SPIM) and light-sheet imaging (LSI), including the use of optical lattices or quasi-lattices (LLSI), have enabled substantial gains against photobleaching and toxicity in optically sectioning cellular or multicellular specimens^1–9^. Conversely, quantitative-phase imaging (QPI) retains low phototoxicity and confers label-free recognition and number-density quantification of single cells and their organelles^10–14^. These two imaging modalities offer complementary advantages. On the one hand, LSI (or LLSI) registers the 4D dynamics of fluorescent proteins with high spatiotemporal resolution and minimal physiology perturbation; QPI, on the other hand, unravels the number-density of cells (and their organelles) that is enthalpically more representative of metabolism than size, as determined by conventional, volumetric, microscopy^12^. Further, QPI’s capability of label-free organelle recognition (e.g., nuclei, mitochondria, lipid droplets) via direct thresholding of the innate refractive index variations^11,12^ or deep learning^15–17^ offers two additional advantages. First, it improves wavelength-multiplexing by freeing spectral channels that would otherwise be occupied by fluorescent reporters targeting specific organelles. Second, it can potentially improve the precision of organelle localization, given that gene-encoded fluorescent reporters can display binding bistability between organelles, such as Glycerol-3-phosphate acyltransferase 4 (GPAT4) decorating both the endoplasmic reticulum and lipid droplets (LDs)^18^.

Integrating QPI with LSI (or LLSI) requires configurations that: (a) are compatible with conventional sample mounting techniques; and (b) do not disrupt image acquisition in either modality. While QPI is compatible with most sample mounting techniques and microfluidics, LSI and LLSI generally require practices that are atypical to common cell culture techniques (e.g., cells embedded in agarose tubes) or are prone to contamination (e.g., objectives dipped into the sample chamber). Regarding undisrupted image acquisition, this is particularly pertinent to QPI since it relies on the interference between the transmitted field through the cell (signal) and a reference field^14^. Any photon losses or spatial frequency limitations to both fields would be detrimental to the fidelity and resolution of the reconstructed image.

Undisrupted QPI is challenging in most LSI (or LLSI) configurations utilizing two orthogonal objectives in close proximity due to the resulting obstructions in the signal or reference paths^3,4^. Single-objective configurations can potentially alleviate any spatial frequency or energy losses and enable compatibility with common sample-mounting techniques. To this end, several single-objective configurations have been recently reported, with some enabling extremely large fields-of-view (FOVs)^19–22^; however, approaches that enable submicron (planar/axial) resolution, such as epi-illumination SPIM or highly inclined and laminated optical sheet (HILO)^6–8^, typically achieve only a fraction of the field-of-view (FOV) endowed by QPI (for example the FOV reported in^8^ with an illumination beam of a 70 μm diffraction-free propagation length is approximately 5 × less than what would be possible with QPI). Inevitably, this FOV discrepancy at submicron (or subcellular) resolution levels can yield significant differences between the throughput rates of QPI and LSI (or LLSI), which can be detrimental to statistically significant investigations at the single-cell level.

**Figure 1:**
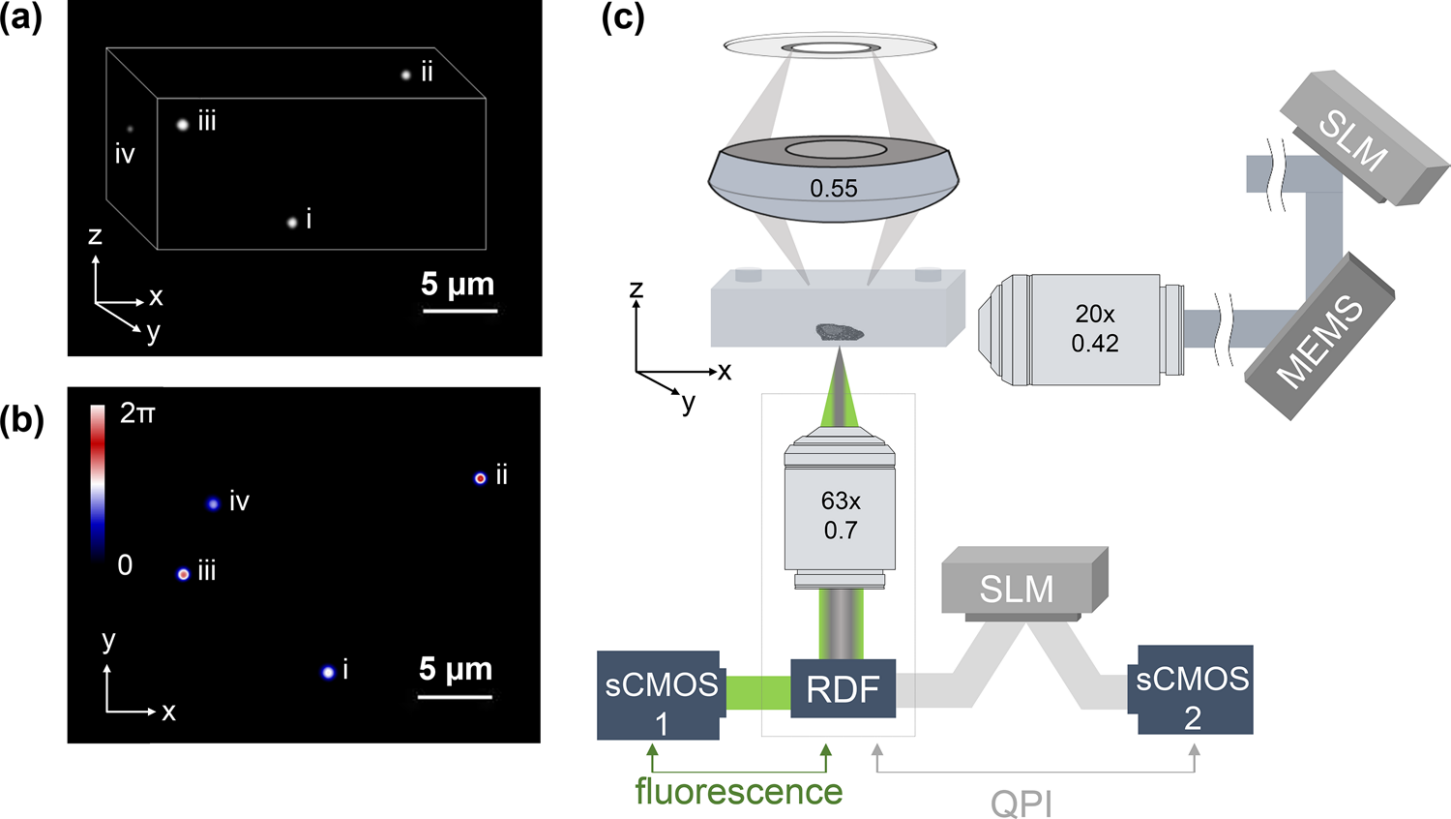
(**a**) A representative 3D fluorescent image of 500 nm diameter particles using Airy beam illumination after deconvolution. (**b**) The same particles as in (**a**) displayed by QPI; inset depicts the scale bar in radians. (**c**) Schematic illustration of the imaging set-up comprising of two objectives arranged orthogonally, one for detection (63x/0.7) and one (20x/0.42) for guiding the Airy beam to a sample enclosed in a microfluidic system; the Airy beam is generated by a spatial light modulator (SLM) and scanned in the *y* direction. A 0.55-NA condenser guides the white illumination on to the sample, and the transmission is encoded by a second SLM to reconstruct the optical-phase image. RDF stands for reflecting dichroic filter, which directs the fluorescent signal to sCMOS_1_ and the transmitted white light to a second SLM and sCMOS_2_*.*

Despite major recent advances in QPI and LSI (or LLSI), these two modalities rely on disparate hardware configurations and sample-mounting techniques. These limitations hinder integrative QPI and LSI imaging and, thus, key live-cell imaging investigations at the single-cell level. One such example pertains to delineating the stochastic effects in gene and metabolic networks^23^, where the former can be deciphered by gene-encoded fluorescent markers and the latter precisely quantified by QPI. Here, we report an integrative imaging system that acquires QPI and LSI images of the same targets (Fig. 1a,b) with identical FOVs. Further, this integrative imaging system exhibits no disruptions to the image acquisition process of either imaging modality, compatibility with conventional sample mounting techniques and microfluidics, and open access software for image acquisition and processing. We detail the design and performance of this integrative imaging system, along with a representative bioimaging application in the following section.

## Results

### Design and assembly

The reported integrative imaging system combines spatial light interference microscopy (SLIM) for QPI with an Airy beam light-sheet for 3D fluorescent imaging on to a standard inverted microscope (Fig. 1c). Specifically, SLIM operates by illuminating the sample in a Koehler configuration through a high numerical aperture condenser (NA 0.55, 2.8 cm working distance) and guiding the sample transmission via an objective (63x/0.7) onto a spatial light modulator (Fig. 1c)^24^. The latter is positioned at a conjugate image plane from a standard camera port and applies additional phase-delays to the non-diffracted wavefront (background) with respect to the diffracted wavefront (see “Methods” for more details). At this configuration, SLIM reconstructs quantitative-phase images of approximately 200 × 200 μm^2^ areas and planar resolution of 0.641 ± 0.006 μm (mean ± standard error, n = 25 beads with a 500 nm diameter). SLIM’s sensitivity and resolution has been previously shown to effectively quantify the growth kinetics of single *Escherichia coli* and mammalian cells^25^ and nutrient allocation between growth and lipid production in single yeast cells^12^.

**Figure 2:**
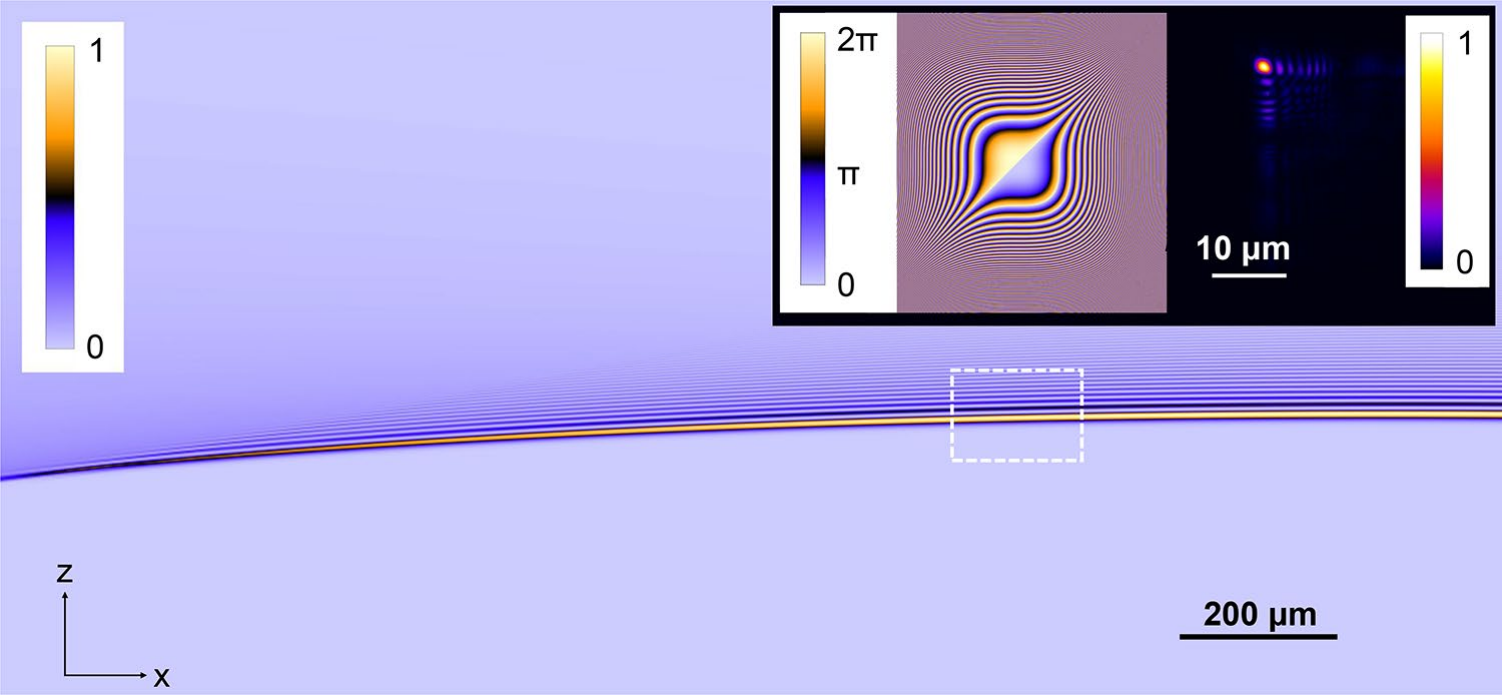
Simulated data for the Airy beam illumination under the experimental conditions, with the dotted square denoting the imaging region; *insets* display the SLM mask employed in these experiments and the experimental Airy beam illumination pattern after the illumination objective.

To integrate QPI with LSI of identical imaging areas and inspired by previous demonstrations^5^, we implemented a self-accelerating Airy beam^26^ for fluorescent light-sheet excitation. The set-up for generating the Airy beam (further detailed in Fig. S1 and in “Methods” ), consisted of a second spatial light modulator that exhibited an appropriate cubic phase mask (Fig. 2). This mask was imaged to the back focal plane of an illumination objective (20x/0.42) with a ~ 0.8 × overall magnification. The illumination objective was positioned orthogonally to the detection objective (Fig. 1c), with the latter being the same (63x/0.7) for both QPI and Airy-LSI.

Guided by a computational model relying on the Fast Fourier Transform Beam Propagation Method (see “Methods” for more details), we adopted an Airy beam with a cubic phase mask exhibiting a α = 5.38 mm^-3^ scaling parameter, thus, varying from − 84 π to + 84 π over a 10 mm range (Fig. 2). This phase mask bestowed an Airy beam with a diffraction-free path (along the *x-axis* in Fig. 1c) that was comparable to the dimensions of the SLIM image. Denser phase modulation further increased the diffraction-free path; however, this approach also positioned the side-lobes of the vertical structure of the Airy beam farther from each other, hindering the utility of high NA objectives.

Importantly, the set-up implemented a long working distance (20 mm) illumination objective and a custom-made 3D microscope stage. The long working distance objective enabled fluorescent excitation without obstructing QPI’s Koehler illumination. The custom stage was equipped with a piezo-electric module to vertically scan the sample with respect to the plane of illumination to enable the 3D image reconstruction. The stage was also open-ended on one side (Fig. S2), enabling complete optical access to the side-plane (*yz* plane in Fig. 1c) of the sample. Optical access was further facilitated by tailored microfluidic systems. These microsystems were fabricated via the combination of UV and soft lithography in a polymer that was index-matched to water and exhibited low side-wall surface roughness using (Fig. S3 and “Methods”). In essence, index matching to water rendered the microfluidics invisible to the fluorescent excitation, thus, eliminating optical aberrations at the polymer-water interface. The bottom wall of the microsystem (*xy* plane in Fig. 1c) and its side wall (*yz* plane in Fig. 1c) were 400 μm and 800 μm thick, respectively. These dimensions enabled imaging cells at the bottom surface of the microsystem without any distortion to the Airy beam^27^. This is because the vertical dimension of the Airy beam (i.e., the extend of the beam’s intensity along the *z-axis* in Fig. 1c) was significantly smaller than the thickness of the bottom wall of the microfluidic system. In turn, this enabled optical access to the sample without the illumination beam crossing the refractive index boundary at the air-polymer interface at the bottom wall of the microsystem. This finding is supported by the experimental results presented in Fig. S4.

### Performance

**Figure 3:**
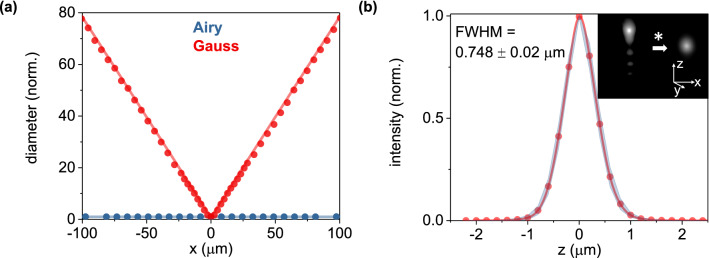
(**a**) Experimental data of the Airy and Gaussian beam propagation and diffraction in a uniformly fluorescent sample; dots illustrate the experimental data and solid lines the simulation results of the approximated beam diameter; the y-axis is normalized to the minimum value of each beam. (**b**) Full-width half maximum (FWHM) along the *z-axis* of 25 fluorescent particles (500 nm in diameter) denoting the axial resolution; the red data points depict the experimentally determined mean values and the blue shaded areas the 95% confidence intervals, while the legend notes the average (± s.e.) of the 25 observations; inset depicts a representative 3D view of particle before (left) and after (right) deconvolution.

In agreement with the computational model, the propagation invariant intensity property of the Airy beam enabled a diffraction-free path (*x-axis* in Fig. 1c) of approximately 200 μm, as visualized in a uniformly fluorescent polymer at a wavelength of 488 nm (Fig. 3a). This value was considerably larger than the respective path of a Gaussian beam at the same wavelength, which increased linearly in diameter by almost two orders of magnitude for the same propagation distance (Fig. 3a). To form a 2D image, the Airy beam was scanned with a MEMS mirror over a 200 μm range (*y-axis* in Fig. 1c). As such, we attained a 200 × 200 μm^2^ imaging area for LSI that matches exactly that of QPI. This imaging area surpasses previous demonstrations utilizing optical lattice configurations^4^ (0.37 μm axial resolution) and propagation invariant Bessel beams in an epi-illumination SPIM format (0.44 μm axial resolution by not considering the side-lobes)^8^, and smaller (by ~ 0.6 ×) than previous Airy beam demonstrations (0.86 μm axial resolution^5^). For comparison, the field-of-view using a Gaussian illumination beam in our set-up was approximately 200 × 10 μm^2^.

To experimentally determine the resolving power of the Airy LS system along the z-axis (Fig. 1c), we employed 500 nm diameter fluorescent particles embedded in a non-scattering matrix. The full-width-at-half-maximum (FWHM) along the z-axis was 0.748 ± 0.020 μm (mean ± standard error, n = 25, Fig. 3b). We obtained this value after deconvolution to remove the transverse structure of the Airy beam, using an experimentally determined 3D point-spread-function and the Richardson-Lucy algorithm (see “Methods” for more details). This value is slightly higher than the system’s planar resolution (determined using 200 nm diameter beads at 0.523 ± 0.021 μm, mean ± standard error, n = 25, Fig. S5), enabling *quasi*-isotropic 3D imaging. Further, our determined lateral resolution was moderately higher than previous reports using an Airy beam light-sheet^5^. We attribute this improvement to the implementation of a higher NA detection objective that enabled the collection of the main Airy lobe as well as 3 side lobes (Fig. 3b, inset). Importantly, the axial resolution was more than half of the diameter of the main lobe of the Airy beam. This suggests that, in agreement with previous demonstrations^5^, the transverse structure of the Airy beam is accounted for by deconvolution and contributes positively to the imaging process.

### Multivariate bioimaging

To demonstrate the applicability of the reported integrative system, we performed a representative live-cell, multivariate imaging investigation in microfluidics using the oleaginous yeast *Yarrowia lipolytica*^28–31^. We selected this model system for two reasons. First, *Y. lipolytica* cells exhibit overall dimensions (ellipsoidal volume with typically a 5.3 ± 0.4 μm major axis, mean ± standard error, Fig. S6) that are challenging for LSI. Second, *Y. lipolytica* represents an important tractable model system for the production of 2nd generation biofuels^28^. In this context, *Y. lipolytica* has been shown to accumulate increased amounts of triacylglycerides, a biodiesel precursor, within a cytosolic organelle termed the lipid droplet (LD)^32–34^. In addition to biotechnology applications, LDs are also central in cellular metabolism and energy homeostasis^35–37^, disease^38–40^, as well as protein, chromatin component and transcription factor trafficking^41–44^.

For imaging, we collected a demonstrative subpopulation of a *Y. lipolytica* culture expressing a GFP-tagged ergosterol 6 enzyme (*erg6*, YALI0F08701g^45^, see “Methods” for more details on the strain) growing in batch. We collected cells at 48 h and 96 h, two timepoints that correspond to low and high lipid content, respectively^12^. Subsequently, we introduced the cells into a microfluidic system (“Methods” ) and allowed them to sediment to the bottom surface.

**Figure 4:**
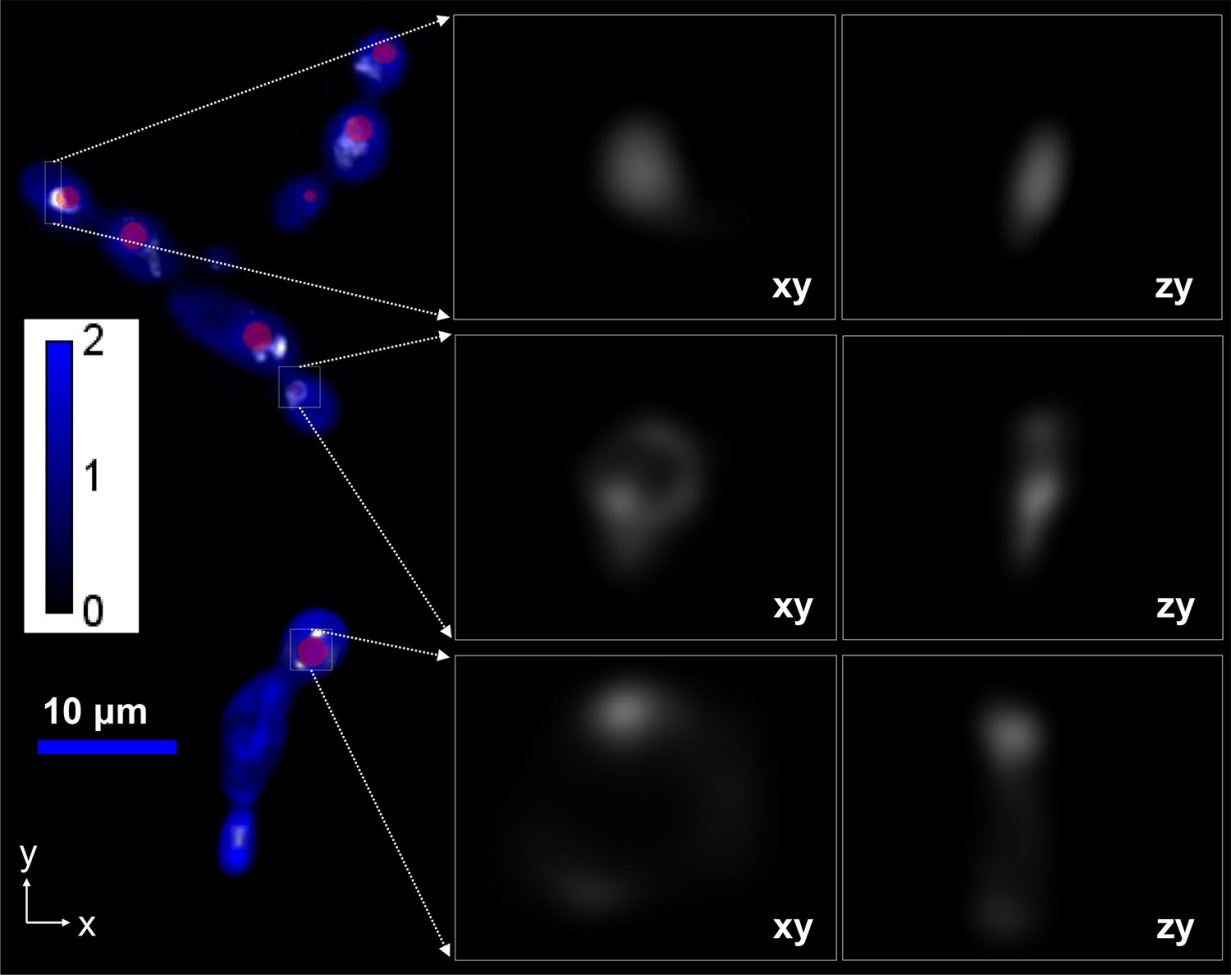
(**a**) Maximum projection (*xy* plane) of a *Y. lipolytica* Airy-QPI integrative image; *blue* denotes the QPI image (scale bar in radians), *red* localizes the LDs (i.e., the phase-thresholded masks), and *white* denotes the GFP-*erg6* fluorescence; on the right, select magnified areas of *erg6* are displayed in the *xy* and *yz* planes with each box size being ~ 5 × 5 × 5 μm^3^.

Critically, QPI enabled the segmentation of single-cells without any dedicated computational approaches or staining procedures, but rather taking advantage of the cells’ higher optical-phase delay than the background^46^. Similarly, we segmented the LDs of individual cells in QPI by relying on their higher optical phase-delay than the cell cytosol (Fig. 4), without any additional staining^12,47^. Within each segmented cell, we quantified the 3D distribution of fluorescent *erg6* enzymes using the Airy LSI at a 488 nm excitation wavelength (Fig. 4).

**Figure 5:**
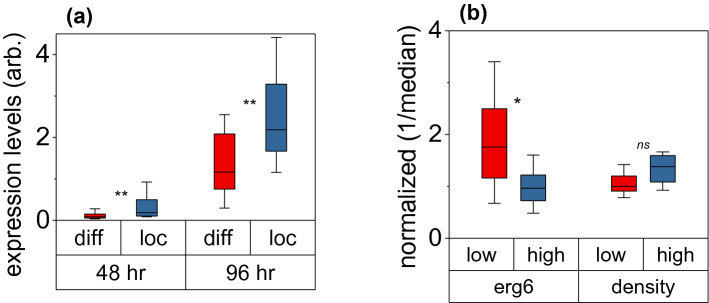
(**a**) Box-plots comparing the *erg6* expression levels for localized (*blue*) and diffusive (*red*) states at two different time points: 48 h (n = 61) and 96 h (n = 55); box-plots represent the 25th and 75th percentiles and whiskers the 10th and 90th percentile; asterisks denote statistically significant differences between distributions (Mann–Whitney Test, U = 100, p = 0.01 for 48 h and U = 150.5, p = 0.001 for 96 h). (**b**) Similar box-plots as in (**a**), comparing the *erg6* expression levels and dry-density for cells exhibiting low (less than 10%) and high (greater than 10%) coverage of the LD surface area by *erg6*; the *y-axis* is normalized with respect to the expression and density median values of the population, while asterisks denote statistically significant differences between distributions (Mann–Whitney Test, U = 64, p = 0.03 for *eg6* expression and U = 18, p = 0.07 for cellular dry-density).

A comparison between the *erg6* distribution with the LD location unmasked two unexpected forms of spatial heterogeneity. In the first, *erg6* either localized in the vicinity of LDs or displayed diffusive behavior (Fig. 4). By diffusive, we specifically refer to states where *erg6* does not localize in the vicinity of an LD, but potentially to the endoplasmic reticulum^48^ or a vacuole^49^. For the latter specifically, cells grown under nutrient limiting conditions can switch from growth and division to carbon storage and autophagy. The process of autophagy can consume the contents of organelles^12,49,50^, which can be a potential source for the dispersed *erg6* signal. Further quantitative analysis revealed that the heterogeneous occupancy of diffusive and localized states occurred both at the early and late stage of lipogenesis, characterized by a statistically significant dependence on the *erg6* expression levels (Fig. 5a). Specifically, cells with overall higher *erg6* expression levels were more likely to exhibit localized *erg6* in the vicinity of an LD.

The second form of spatial heterogeneity pertained particularly to *erg6* localized states. Focusing on the 96 h timepoint, we observed that enzymes did not uniformly decorate the 3D surface of LDs, but rather exhibited clusters that offered only partial coverage (Fig. 4). Using the 3D resolving capability of the Airy LSI, we quantified the percentage of the LD surface area that was in contact with *erg6*. In this context, we observed that the low relative contact areas exhibited increased *erg6* expression levels, while conversely, high contact areas exhibited lower expression levels of *erg6* (Fig. 5b). In other words, cells with overall increased *erg6* expression levels were statistically more likely to exhibit reduced contact areas between LDs and *erg6* (Fig. 5b). Interestingly, similar statistical evidence between the cellular dry-density (determined by the cell’s optical phase, see “Methods” ) and LD coverage was not found (Fig. 5b). The latter indicates that cells stochastically overproducing biomass building blocks (e.g., proteins, ribosomes, DNA, lipids) do not regulate specifically the contact area between *erg6* and LDs, and possibly the spatial heterogeneity of ergosterol biosynthesis. Critically, the increased cellular density did not correlate with *erg6* expression levels, suggesting that increased production of biomass does not gratuitously lead to *erg6* overexpression^51^.

## Discussion

Single-cell biology has led to a plethora of unexpected discoveries, primarily catalyzed by advances in optical imaging^52–56^, as well as sequencing and metabolomics^57–59^. In the context of optical imaging, the advent of SPIM, LSI and LLSI has greatly advanced our ability to probe dynamic and structural cellular phenotypes at unprecedented imaging speeds and phototoxicity levels^1–9^. Low phototoxicity is also bestowed by QPI, which additionally informs about the location, size, and dry-density of cells and their organelles^10–17^. Importantly, dry-density offers insight that is enthalpically more pertinent to cellular physiology and metabolism than what is possible by volumetric bioimaging, including LSI ^12^.

Here, we reported the fusion of these two imaging modalities on to a standard inverted microscope. By relying on a self-accelerating Airy beam illumination pattern, this integrative system exhibited identical fields-of-view in each modality without any disruptions to their respective optical paths. Importantly, this design is compatible with microfluidics that alleviate some of the stringent culture and sample preparation techniques required by common LSI and LLSI configurations. Further, the described integrative design is compatible with most QPI methods, including holographic tomography and appropriate methods for thick and multiply scattering samples^60,61^, open-source software^62,63^, and alternative microscope frames, making it accessible to the broader scientific community, including non-specialists.

As a representative example, we applied the reported integrative imager to a microorganism that is challenging to image due to its small dimensions. In this context, we discovered that clonal cells can exhibit two distinct forms of spatial heterogeneity. Specifically, we observed that enzymes participating in ergosterol biosynthesis localized heterogeneously both in the cytosolic milieu and around the LDs. We also observed that, when localized, *erg6* decorates heterogeneously the LDs. These two forms of spatial heterogeneity suggest that ergosterol biosynthesis (and possibly degradation) is likely also spatially heterogeneous. Such forms of heterogeneity not only indicate that LD recognition solely from the GFP signal of *erg6* can be limiting, but also that cellular noise affecting the levels of *erg6* expression, may also impact the compartmental localization of metabolic reactions. This observation raises further questions about the underlying metabolic costs, efficiencies, and evolutionary consequences of metabolic compartmentalization^64^. This live-cell imaging experiment represents one example of how the present integrative imaging system can be applied to single-cell biology investigations, namely: the application of QPI to inform about organelle location in a label-free fashion and cell metabolism via the enthalpically relevant metric of dry-density, and Airy LSI to inform about 3D protein dynamics.

## Methods

### Optical setup

The integrative imaging setup is detailed in Fig S1. We employed a laser source (EXLSR-488C-200-CDRH, Spectra Physics, 200 mW, 488 nm) for fluorescence excitation. The beam was expanded to a 10 mm diameter using a spatial filter (910A, Newport) comprising of a 25 μm diameter pinhole and an objective (10x/0.25 NA, Wild Heerbrugg), and subsequently collimated with a converging lens of 25 cm focal length. A half-wave plate (WPHSM05-488, Thorlabs) was installed before the Spatial Light Modulator (MSP 1920-400-800-HSP8, Meadowlark Optics). The SLM displayed a cubic phase mask that was generated in Matlab (Mathworks), with the 0 and 2π phase levels corresponding to 0 and 255 grey-levels, respectively. A 4f system (f_1_ = 75.6 cm and f_2_ = 40 cm lenses) was installed with f_1_ image conjugated to the SLM (i.e., the SLM was placed at the back-focal plane of f_1_) and f_2_ conjugated to a laser scanner (i.e., the laser scanner was placed at the focal plane of f_2_). The scanner was assembled within a 30 mm cage system, and comprised of a second 4f system (f_3_ = 3.5 cm and f_4_ = 1.2 cm), a 2D MEMS mirror (2.8 mm diameter, Mirrorcle Technologies), a scan lens (f_5_ = 7.5 cm), a tube lens (f_6_ = 30 cm), and a large working distance illumination objective (20x/0.42, Mitutoyo). The long focal-length tube lens was specifically employed to accommodate the extend of the microscope frame along the *y-axis* (Fig. 1c and Sup. Figure 2). The scanner was positioned on a custom-made 3D stage with a 5 cm travel range in all three axes, 1 μm resolution in the vertical axis (*z-axis* in Fig. 1c) and 10 μm planar resolution (*xy* plane in Fig. 1c). The 3D stage enabled us to precisely align the focused Airy beam with the focal planes of the detection objective (63x/0.7, PH2, Leica) and the condenser of a standard inverted microscope (DMi8, Leica). The microscope was equipped with a focus stabilization system, as well as automation in the objective turret and filter wheel position. 3D fluorescent images were captured by a scientific CMOS camera (ORCA-Flash 4.0, Hamamatsu) connected to one port of the microscope using the MicroManager ASIdiSPIM plugin. A spatial light interference microscopy (SLIM) set-up was installed on the second microscope port to relay the conjugate image plane onto a second sCMOS camera (ORCA-Flash 4.0, Hamamatsu) using a 4f system (15 cm focal lengths for both lenses), as previously described^20^. A second SLM (STD 512-450-850-ExtFlip, Meadowlark Optics) was positioned at the Fourier plane of the first lens of the 4f system to modulate the phase difference between the scattered and un-scattered components of light at increments of π/2, π, 3π/2 and 2π. Similar to conventional phase contrast microscopy, the scattered and un-scattered wavefronts were separated by applying hollow cone illumination using an illumination condenser (in Koehler configuration) equipped with the appropriate annulus (PH2). Under these illumination conditions, only the scattered light deviates from the illumination cone and, thus, is projected on to a different location of the SLM. Using this approach, we acquired quantitative-phase images in an automated fashion (CellVista Pro, Phi Optics) through the same detection objective, informing about the relative phase delay of the cells (scattered wavefront) with respect to the background (un-scattered wavefront). According to previous reports^24^, the minimum detectable phase step is less than 1 mrad with this system.

### Optical alignment

Irises along the optical path (Fig. S1) ensured the excitation laser passed through the center of all optical elements, as well as enabled the selection of the first-order diffraction from the SLM and alignment inspection on a daily basis. Once installed, our optical system remained stable for months, requiring only minor mirror adjustments every few days. The active SLM pattern was aligned with the excitation beam using a CMOS camera (acA3800-14um, Basler). During installation, we ensured that the microscope stage and scanner were properly aligned by coupling the 488 nm beam to a single mode fiber (MBT613D, Thorlabs) and interfacing it with the scanner’s cage through a fiber-port collimator (PAF2P-11E, Thorlabs). By inspecting the excitation beam profile at the image plane, we made the necessary adjustments in the scanner and microscope position until the illumination and detection planes overlapped. Subsequently, we removed the fiber and aligned the free-space 488 nm beam with respect to the input and output ports of the scanner using an alignment target (CPA1, Thorlabs) and a CMOS camera mounted on the microscope stage (acA3800-14um, Basler). The Airy beam quality was inspected both with the stage-mounted CMOS camera, as well as the sCMOS camera connected to the microscope port. For the latter, we employed both a custom-made 90^ο^ reflector on the microscope stage or a fluorescent polydimethilsiloxane (PDMS) sample.

### Custom microscope stage

A custom-made microscope stage was employed to position the sample and scan it in all three directions for imaging (Fig. S2). The stage comprised of a piezoelectric system (IPZ-3150, Applied Scientific Instrumentation) with a 2.2 nm resolution and 150 μm total travel range. The piezo stage was integrated with two linear stages (LS-50, Applied Scientific Instrumentation), each exhibiting 5.5 nm encoder resolution and 50 mm total travel range. For acquisition, we synchronized the piezo stage, linear stages, and 2D MEMS mirror using a controller (TG-1000-8, Applied Scientific Instrumentation) equipped with programmable logic, and communication cards. To enable 3D imaging at a single location, the sample was scanned vertically (i.e., along the *z-axis* in Fig. 1c) using the piezo stage, and the Airy beam planarly (i.e., along the *y-axis* in Fig. 1c) using the MEMS mirror (Fig. S1).

### Data acquisition and processing

Micro-Manager 1.4^62^ and CellVista Pro (Phi Optics)^24^ were used for fluorescent and quantitative-phase image acquisition. A PC (Z8, Hewlett-Packard) equipped with Intel Xeon W-2123 W CPU @ 3.60 GHz processors and 128 GB RAM acquired and temporarily stored raw 3D images. For longer term storage, we transferred all data to a server. Images were analyzed using ImageJ^65^ on a workstation equipped with an Intel Core i7-7820X CPU @ 3.60 GHz processors and 128 GB RAM. Image deconvolution was performed using the Richardson-Lucy algorithm in the DeconvolutionLab2 ImageJ plugin^63^, using an experimentally determined point spread function (PSF) derived from a 3D stack of 0.2 μm diameter fluorescent particles. 3D image reconstruction was performed using the VolumeViewer plugin (ImageJ).

### Optical model

To guide our experimental approach and validate our results (Fig. 3), we built a numerical model to describe the propagation of the Airy beam. The model considers a monochromatic wave with an electric field component in the $\stackrel{\sim }{E}(x,y,z,t)=E(x,y,z){\bullet e}^{-i\omega t}$ form. This wave satisfies the Helmholtz wave equation ${\nabla }^{2}E+{k}^{2}E=0$, where $k=\frac{2\bullet \pi \bullet n}{\lambda }$ is a wavenumber, $\lambda$ represents the wavelength of the illumination and $n$ the refractive index. The wave’s $E(x,y,z)$ component can be written as a Fourier superposition of plane waves in the following form:

1 $$E\left(x,y,z\right)=\frac{1}{{\left(2\uppi \right)}^{2}}\underset{-\infty }{\overset{+\infty }{\iint }}d{k}_{x}\bullet d{k}_{y}\bullet A({k}_{x},{k}_{y}){\bullet e}^{i\left({k}_{x}x+{k}_{y}y\right)}\bullet {e}^{i{k}_{z}z}$$

where ${k}_{z}=\sqrt{{k}^{2}-{k}_{x}^{2}-{k}_{y}^{2}}$ and $A({k}_{x},{k}_{y})$ is the Fourier spectrum defined by the initial field profile at the input $z={z}_{o}$ through a Fourier transform:

2 $$A\left({k}_{x},{k}_{y}\right)=\underset{-\infty }{\overset{+\infty }{\iint }}dxdyE(x,y,{z}_{o}){e}^{i\left({k}_{x}x+{k}_{y}y\right)}$$

We solved Eqs. (1) and (2) via the Fast Fourier Transform Beam Propagation Method (FFT-BPM) that computes the field profile $E(x,y,z)$ at any z for a known field distribution at ${z}_{o}=0$. As the input field (at ${z}_{o}=0$), we considered Gaussian illumination of the SLM cubic phase, namely: ${E}_{o}=E\left(x,y,z=0\right)={Ae}^{-\frac{{x}^{2}+{y}^{2}}{{w}_{o}^{2}}} {\bullet e}^{\frac{i\bullet \alpha }{3}\left({x}^{3}+{y}^{3}\right)}$ , where $\alpha$ is the scaling parameter of the phase, and ${w}_{o}$ and $A$ represent the width and the amplitude of the illumination, respectively. Utilizing the FFT-BPM algorithm, we propagated the beam from $z=0$ to all individual lenses (i.e., at distances $z={f}_{i}$, with ${f}_{i}$ being the focal length of each lens) described in the optical set-up of Fig S1 and multiplied the field $E\left(x,y,z={f}_{i}\right)$ with the transmission function ${T}_{i}(x,y)$ of each lens. To mimic the propagation through the lens, we used the thin lens approximation with the transmission function ${T}_{i}\left(x,y\right)= {e}^{-\frac{i\bullet \frac{2\bullet \pi }{\lambda }\bullet ({x}^{2}+{y}^{2})}{2\bullet {f}_{i}}}$. The employed algorithm is detailed as follows:

1. Start from the input field at the SLM plane $z={z}_{o}$, given by ${E}_{0}$.
2. Propagate ${E}_{0}$ in free space up to the first lens, placed at ${z}_{1}={z}_{o}+{f}_{1}$:
  $${E}_{01}=IFFT\left\{FFT\left\{{E}_{0}\right\}{e}^{i{k}_{z}{f}_{1}}\right\}$$
3. Compute the field after the first lens as:
  $${E}_{1}={E}_{01}{\bullet T}_{1}$$
4. Propagate ${E}_{1}$ in free space until the second lens, placed at ${z}_{2}={z}_{1}+{f}_{1}+{f}_{2}$:
  $${E}_{12}=IFFT\left\{FFT\left\{{E}_{1}\right\}{e}^{i{k}_{z}\left({f}_{1}+{f}_{2}\right)}\right\}$$
5. Compute the field after the second lens as:
  $${E}_{2}={E}_{12\bullet }{T}_{2}$$
6. Propagate and compute beam after all experimental lenses, up until the last lens, placed at ${z}_{7}={z}_{6}+{f}_{6}+{f}_{7}$:
  $${E}_{67}=IFFT\left\{FFT\left\{{E}_{6}\right\}{e}^{i{k}_{z}\left({f}_{6}+{f}_{7}\right)}\right\}$$
7. Compute the field after the last lens as:
  $${E}_{7}={E}_{67}{\bullet T}_{7}$$
8. Propagate ${E}_{7}$ in free space for another distance $\Delta {z}_{8}$:
  $${E}_{8}=IFFT\left\{FFT\left\{{E}_{7}\right\}{e}^{i{k}_{z}\Delta {z}_{8}}\right\}$$

To overcome memory limitations while maintaining high accuracy, we simplified the model to 2D by neglecting the third dimension. The parameters used in the simulation were: $\lambda =0.488 \mu m$, $n=1$, $A=1$, ${w}_{o}=4.3 mm$, $\alpha =5.38 {mm}^{-3}$, while all focal lengths were identical to the experimental ones listed in Fig. S1 and the illumination objective set at ${f}_{7}=2 cm$. We obtained the final results at a 4 mm range around the focal length of the last lens with a grid size of $N={2}^{20}$, as well as a transverse and longitudinal sampling of $\Delta x=0.2 \mu m$ and $\mathrm{\Delta z}=0.2 \mu m$.

### Optical resolution and PSF characterization

To characterize the axial resolution of our optical system (Fig. 3b), we employed fluorescent particles embedded in an agarose gel. To prepare the gel sample, we mixed 1.5% agarose (Ultra-Pure, Invitrogen) with deionized water and kept the mixture in a convection oven at 80 °C for 45 min until the agarose completely dissolved. Subsequently, fluorescent micro-spheres were added to the gel and mixed thoroughly. For optimal particle density, we diluted the particle solution of 1% solids by approximately 10^4^. The mixture was then poured into a custom holder, in-between two coverslips for 15 min to solidify prior to imaging. Specifically, we employed 0.5 μm and 0.2 μm mean diameter particles (FSDG003 and FSDG004, Bangs Laboratories) to quantify the system’s z-resolution and point spread function, respectively. For both measurements, we employed a 100 μW excitation power after the illumination objective, a 525/50 bandpass filer (Chroma) and a 0.2 μm step size on the piezo stage. To quantify the system’s axial and planar resolution, we averaged n = 25 observations. To determine the system’s PSF, we averaged n = 5 observations and subsequently used the average PSF in the deconvolution algorithms.

### Propagation length and diffraction quantification

To experimentally determine the diffraction-free propagation length of the Airy and Gaussian beams (Fig. 3a), we illuminated a homogeneously fluorescent sample and quantified the beam diameter (approximated by 1/e) through the lateral intensity distribution (along the *y-axis* in Fig. 1c) at various propagation distances (along the *x-axis* in Fig. 1c). The fluorescent sample was prepared in a polydimethylsiloxane (PDMS Sylgard 184, Dow Corning) matrix mixed with the chromophore Lumogen orange (BASF). To this end, we first mixed and degassed the PDMS monomer with its catalyst (10:1 ratio), and subsequently added 10 μL of a Lumogen Orange dichloromethane (HPLC LC–MS grade, OmniSolv) solution at 0.1 mg/ml. The mixture was let to cure for 2 h at 70 °C prior to imaging. The experimental results of the Airy beam propagation shown in Fig. 3a were co-plotted with the FFT-BPM computational results, and the Gaussian results with an analytical expression for the Gaussian beam waist (w) as a function of the propagation distance z ($w\left(z\right)={w}_{0}\sqrt{1+{(\frac{z}{{z}_{r}})}^{2}}$, with w_o_ set at 1.2 μm).

### Strain and growth conditions

The *Y. lipolytica* strain used in this study was generated on an auxotrophic background with decreased non-homologous end joining for targeted DNA incorporation, as previously described^45^. The strain was modified using a superfolder GFP plasmid to endogenously express fluorescent *erg6*, an enzyme in the ergosterol biosynthesis pathway. For the rich YPD medium, we mixed 20 g/L Bacto Peptone (BD), 10 g/L yeast extract (Alfa Aesar), and 20 g/L glucose (Fisher). The defined YSM medium at a C:N ratio of 150 contained 1.7 g/L yeast nitrogen base without amino acids and without ammonium sulfate (BD Difco), 0.69 g/L complete supplement mixture (CSM) without Leucine (Sunrise Science Products), and 1.1 g/L ammonium sulfate (Fisher) and 75 g/L glucose (Fisher). The *erg6* expressing *Y. lipolytica* preculture was stored in YPD agarose (Invitrogen) plates at 4 °C and passed twice in YPD medium (5 ml round bottom polystyrene tubes) for 24 h, with the second passage performed at a 50 × dilution. Subsequently, the YPD culture was centrifuged at 490 × g, and washed in YSM three times, and transferred to 125 ml glass flasks (Corning) containing 20 ml of YSM medium. The flasks were covered with polypropylene closures (Corning) and aluminum foil. Cells were transfered from YPD to YSM at a 0.01 OD. All growth experiments were performed in a shaking incubator (180 rpm) at 28 °C. To perform integrative QPI and Airy LSI imaging, we diluted the growing *Y. lipolytica* culture by 25 × and transferred approximately 10 µL to the microfluidic system and allowed cells to sediment to the bottom surface. Under these conditions, no 3D motion or drift was observed for the cells during image acquisition. We imaged the cells at 48 h and 96 h, corresponding to states of high and low triacylglyceride content^12^.

### Microfluidics

For cell imaging, we employed a polymer microfluidic system microfabricated in a polymer matrix with a refractive index matched to that of water (BIO-133-BP30, My Polymers). The microfluidic system comprised of a rectangular micro-container defined by 800 μm thick vertical sidewalls. This microsystem was first fabricated in PDMS using conventional cast-molding lithography from a patterned SU8 coated Si wafer, and subsequently transferred to BIO-133-BP30 via UV illumination in a mask-aligner (Q4000-4, Quintel Corporation). Once cells were introduced into the micro-container with a pipette, the microsystem was enclosed with a coverslip coated with a 400 μm thick film of the same polymer. Fluid exchange was possible during cell loading or alternatively via external tubes. To inspect the surface quality of the microsystem’s side-wall (Fig. S3), we positioned it on top of a coverslip and we imaged it in a backscattering geometry (similar to the principle of Fizeau interferometry) using broadband illumination (500–700 nm), a 4 × magnification objective and a sCMOS camera. This set-up enabled the visualization of the interference fringes between the polymer and the glass coverslip and, thus, the indirect assessment of surface quality.

### Cell imaging

Select areas were first imaged by QPI and subsequently by the Airy beam at 488 nm. Stacks of 200 planes were acquired at a 0.2 μm step size. Single-cells were localized via maximum projection of the QPI image, and segmented to localize the cell and LD contours via direct phase-thresholding at different levels in ImageJ. Using the cell’s 2D segmented region of interest (ROI), we quantified cell area and the average optical phase (<Φ>) and subsequently the cell dry-density at a wavelength of λ = 500 nm and a refractive index increment (dn/dc) of 1.85 × 10^−4^ m^3^ kg^−1^ using the following equation:

3 $$\rho =\frac{\lambda }{2\bullet \pi \bullet \frac{dn}{dc}}<\Phi >$$

The expression levels of *erg6* per cell contour were quantified by subtracting the cell’s average fluorescent intensity from the extracellular background. The % coverage of the 3D LD surface by *erg6* was quantified by counting the *erg6* fluorescence voxels (i.e., 3D pixels with a total volume of 2 × 10^–3^ μm^3^) that were in contact with the LD, the 3D location of which was estimated from the LD diameter.

### Statistics

Mann–Whitney tests were performed in OriginPro (Origin Pro 2017 64-bit, OriginLab).

## Supplementary information

Supplementary Information.
