# Supplementary material for: Integrative quantitative-phase and airy light-sheet imaging
Source: Sci Rep. 2020 Nov 19;10:20150. doi: 10.1038/s41598-020-76730-x (PMC7678854; doi:10.1038/s41598-020-76730-x)
Supplement: Supplementary file 1 — Supplementary Information. [file 41598_2020_76730_MOESM1_ESM.docx]

**Supplemental Figures for:**

**Integrative Quantitative-Phase and Airy Light-Sheet Imaging**

N. R. Subedi, P. S. Jung, E. L. Bredeweg, S. Nemati, S. E. Baker, D. N. Christodoulides, A. E. Vasdekis

**Supplementary Figure 1**

**
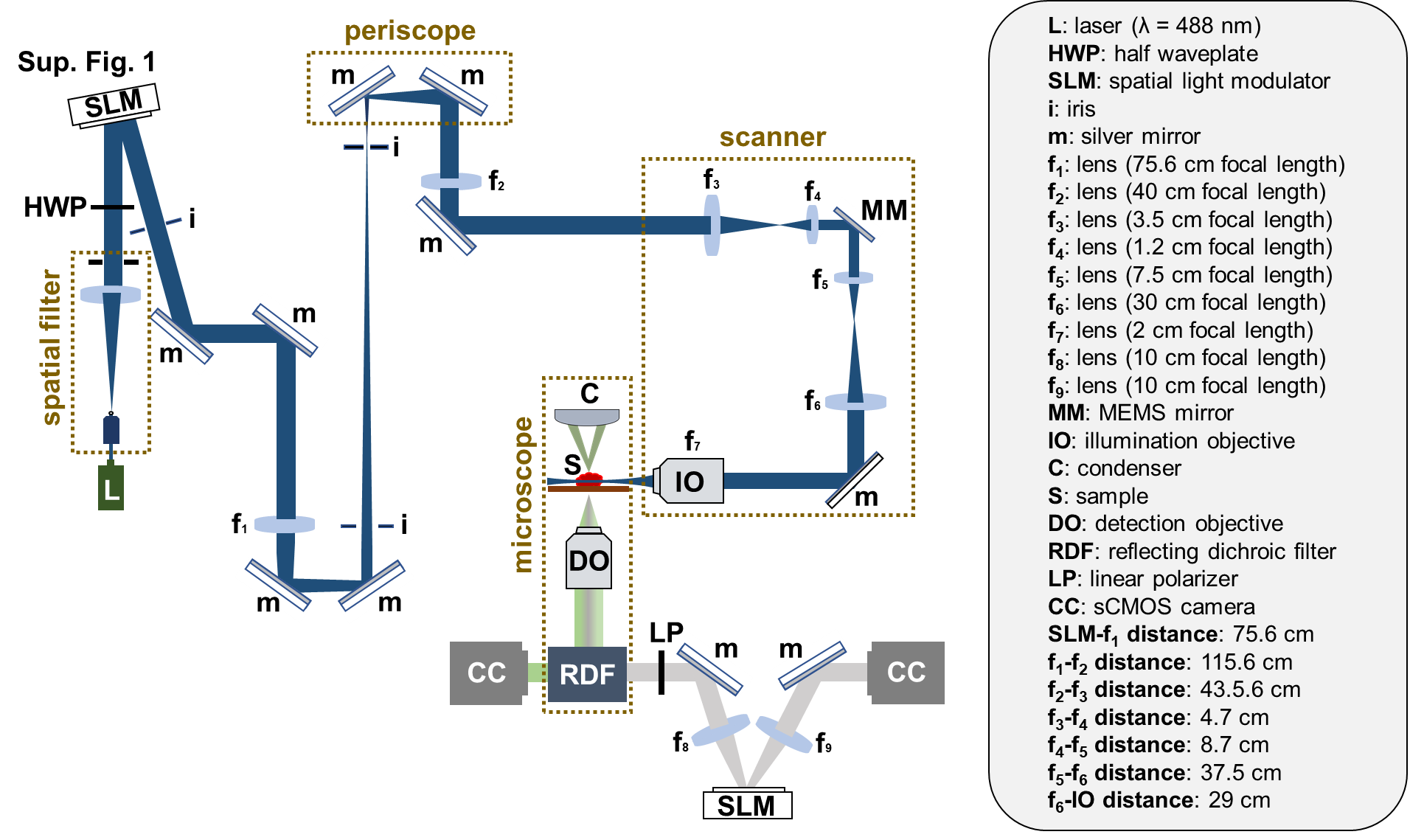
**

**Fig. S1:** The optical set-up enabling integrative Quantitative Phase and Airy light-sheet imaging. Table lists all the components used, along with the focal lengths and positions of all lenses.

**Supplementary Figure 2**

**
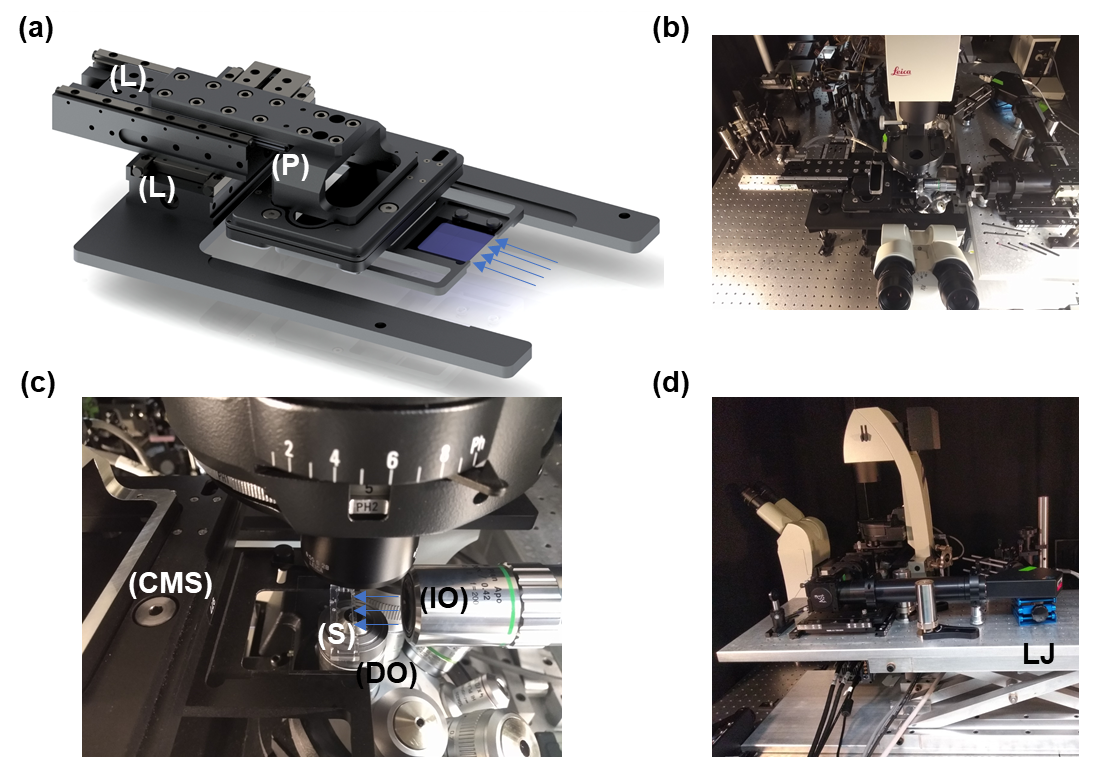
**

**Fig. S2:** **(a)** The custom-built stage that is compatible with a standard inverted microscope and comprises of two linear stages (**L**), a piezo stage (**P**) and a sample holder location that enables sample illumination by an accelerating Airy beam in the direction noted by the blue arrows. **(b)** Front view of the set-up, including the body of the commercial microscope, laser scanner system, and the custom-built microscope stage illustrated in **(a)**. **(c)** Detailed view of the assembly showing the illumination (**IO**) and detection (**DO**) objectives, the custom-built microscope stage (**CMS**) and sample (**S**) location; blue arrows denote the propagation direction of the Airy beam. **(d)** Side-view of the custom lab jack (**LJ**) bringing the scanner assembly and illumination objective to the height of the sample.

**Supplementary Figure 3**

**
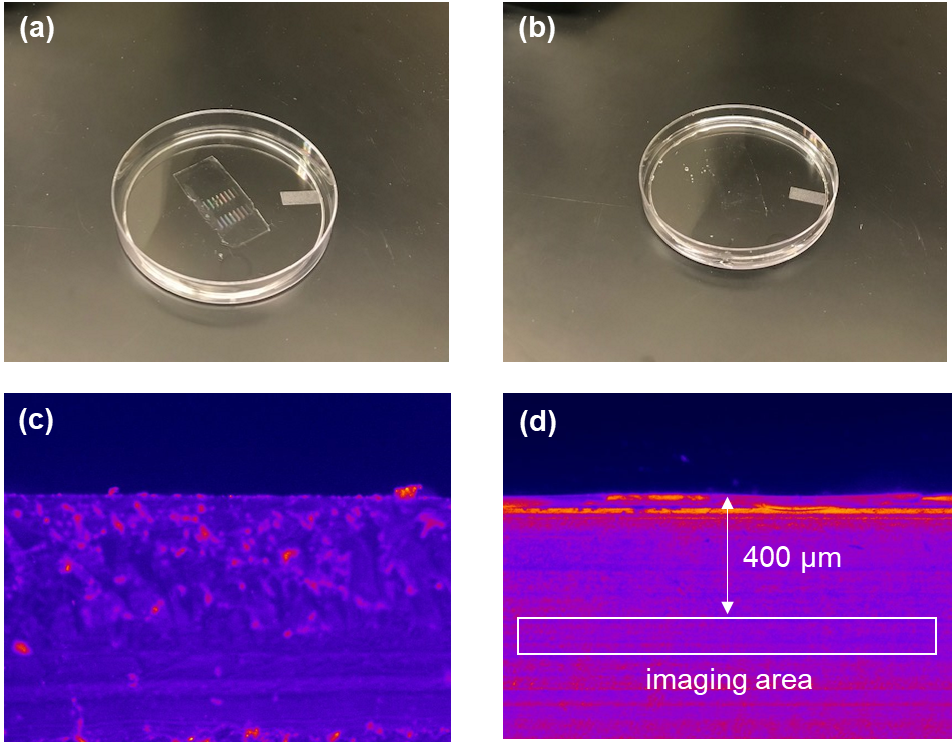
**

**Fig. S3:** **(a)** An imprinted BIO-133-BP30 film with an array of 1D diffraction gratings of different periods; the gratings can be readily visualized due to the polymer’s refractive index contrast with air. **(b)** The same polymer immersed in water, essentially rendering the gratings and the polymer itself invisible due to the film’s index matching with water. Comparison of the side-wall quality of a (c) PDMS slab (cut with a scalpel) and (d) the BIO-133-BP30 microsystem employed in the live-cell imaging exercises. The images were acquired in a backscattering geometry by placing both polymer pieces on a coverslip, while the white box denotes the imaging area (400 µm above polymer edge). Scattering centers are not observed in the case of BIO-133-BP30 (as in PDMS), while the uniformity of the observed (backscattered) fringes between the polymer surface and the glass coverslip suggest high surface quality.

**Supplementary Figure 4**

*
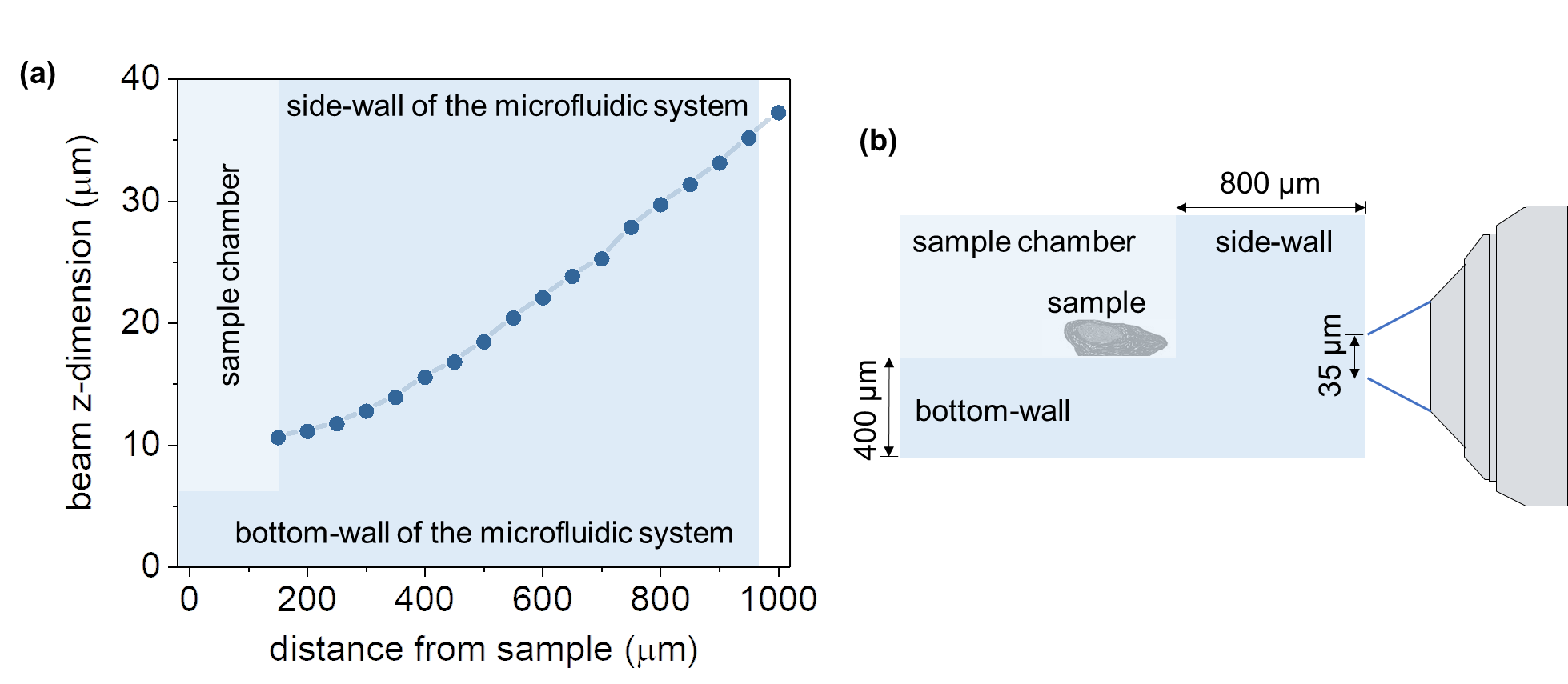
*

**Fig. S4**: The evolution of the Airy beam along the *z-axis* (see **Fig. 1c**) as a function of its propagation distance (*x-axis* in **Fig. 1c**); the graph represents the experimental data that were determined by translating a CMOS sensor with respect to the illumination objective (in the absence of the microfluidics). Blue shaded area denotes the side- and bottom-walls of the microfluidic system; light blue area indicates the sample chamber. The measurement indicates that, upon meeting the polymer microsystem, the vertical dimension of the Airy beam (~35 μm along the *z-axis*) was smaller than the bottom-wall of the microsystem itself (400 μm) by more than one order of magnitude. While the beam’s intensity distribution may change upon entering the polymer matrix, we do not anticipate its diameter to increase by 10-fold, which would force the beam to cross the air-polymer interface at the bottom wall of the microsystem. **(b)** Cartoon schematic of the graph shown in **(a)** comparing the vertical extent of the illumination beam before entering the microfluidic system with respect to the dimensions of the side- and bottom-walls of the microsystem.

**Supplementary Figure 5**


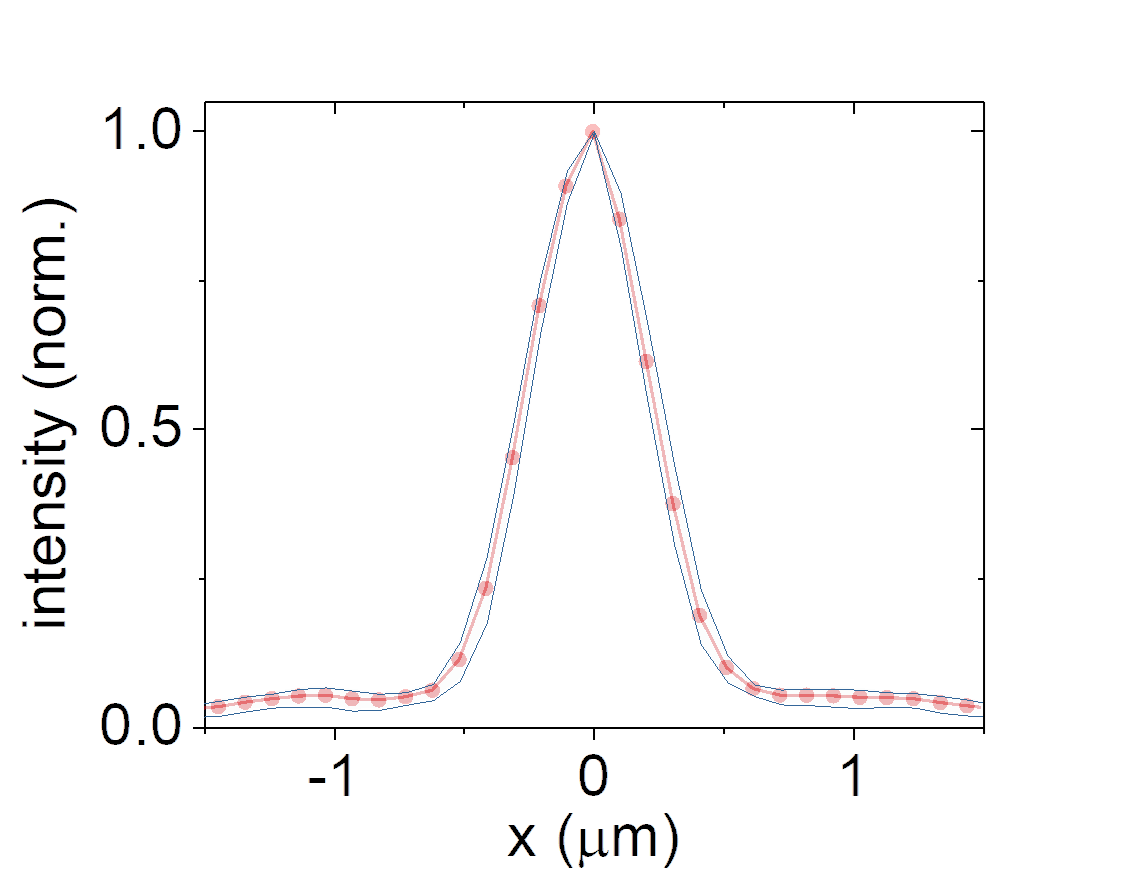


**Fig. S5:** Full-width half maximum (FWHM) along the x-axis of 25 fluorescent particles (200 nm in diameter) yielding at 0.523± 0.021 μm value (mean ± standard error); the solid red line depicts the mean and blue shaded areas the 95% confidence intervals of the mean.

**Supplementary Figure 6**

**
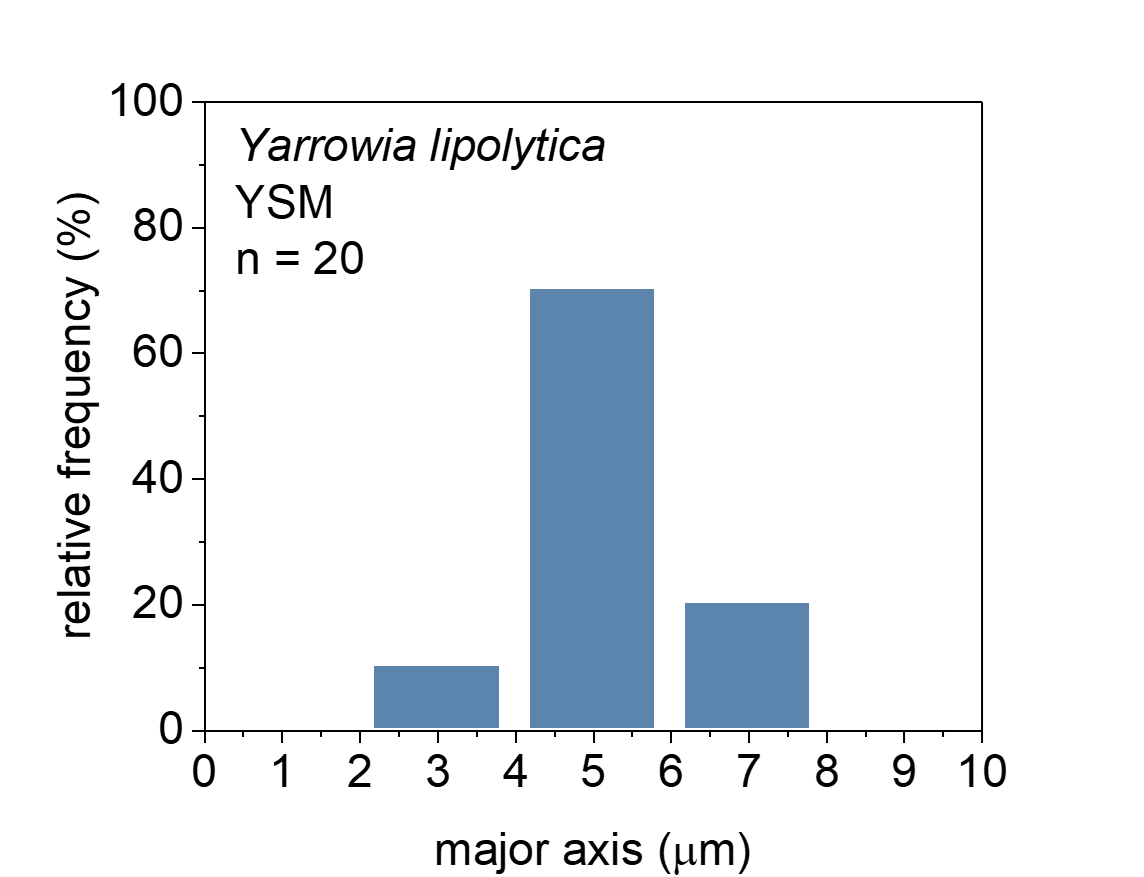
**

**Fig. S6:** The major axis of *Yarrowia lipolytica* grown at a defined YSM medium with a C:N ratio of 150.
